# New Nitride Nanoceramics from Synthesis-Mixed Nanopowders in the Composite System Gallium Nitride GaN–Titanium Nitride TiN

**DOI:** 10.3390/ma14143794

**Published:** 2021-07-07

**Authors:** Mariusz Drygaś, Katarzyna Lejda, Jerzy F. Janik, Klaudia Łyszczarz, Stanisław Gierlotka, Svitlana Stelmakh, Bogdan Pałosz

**Affiliations:** 1 Faculty of Energy and Fuels, AGH University of Science and Technology, al. Mickiewicza 30, 30-059 Kraków, Poland; madrygas@agh.edu.pl (M.D.); kkapusta@agh.edu.pl (K.L.); 2 Faculty of Materials Science and Ceramics, AGH University of Science and Technology, al. Mickiewicza 30, 30-059 Kraków, Poland; lyszczarzk@student.agh.edu.pl; 3 Institute of High Pressure Physics, Polish Academy of Sciences, ul. Sokołowska 29/37, 01-142 Warszawa, Poland; stanislaw.gierlotka@unipress.waw.pl (S.G.); svetlana.stelmakh@unipress.waw.pl (S.S.); bogdan.palosz@unipress.waw.pl (B.P.)

**Keywords:** gallium nitride, titanium nitride, sintering, nanoceramics, Vicker’s hardness

## Abstract

Presented is a study on the preparation, via original precursor solution chemistry, of intimately mixed composite nanocrystalline powders in the system gallium nitride GaN–titanium nitride TiN, atomic ratio Ga/Ti = 1/1, which were subjected to high-pressure (7.7 GPa) and high-temperature (650, 1000, and 1200 °C) sintering with no additives. Potential equilibration toward bimetallic compounds upon mixing of the solutions of the metal dimethylamide precursors, dimeric {Ga[N(CH_3_)_2_]_3_}_2_ and monomeric Ti[N(CH_3_)_2_]_4_, was studied with ^1^H- and ^13^C{H}-NMR spectroscopy in C_6_D_6_ solution. The different nitridation temperatures of 800 and 950 °C afforded a pool of in situ synthesis-mixed composite nanopowders of hexagonal h-GaN and cubic c-TiN with varying average crystallite sizes. The applied sintering temperatures were either to prevent temperature-induced recrystallization (650 °C) or promote crystal growth (1000 and 1200 °C) of the initial powders with the high sintering pressure of 7.7 GPa having a detrimental effect on crystal growth. The powders and nanoceramics, both of the composites and of the individual nitrides, were characterized if applicable by powder XRD, SEM/EDX, Raman spectroscopy, Vicker’s hardness, and helium density. No evidence was found for metastable alloying of the two crystallographically different nitrides under the applied synthesis and sintering conditions, while the nitride domain segregation on the micrometer scale was observed on sintering. The Vicker’s hardness tests for many of the composite and individual nanoceramics provided values with high hardness comparable with those of the individual h-GaN and c-TiN ceramics.

## 1. Introduction

Not found in nature man-made metal and metalloid nitrides have already confirmed their utmost utility in modern technology with a noticeable share in common-day applications including Blu-ray devices and efficient LED laser and light sources [1,2,3,4]. Many of them are semiconductors, and examples include the wide-bandgap semiconductor gallium nitride GaN, E_g_ = 3.4 eV for hexagonal polytype and 3.2 eV for cubic polytype, the narrow-bandgap indium nitride InN, E_g_ = 0.7–0.8 eV, and their intermediate-bandgap binary solid solutions of In_x_Ga_1−x_N with E_g_ = 0.7–3.4 eV and Al_x_Ga_1−x_N with E_g_ = 3.4–6.2 eV [5]. Additional important materials applications dwell on unique sets of properties all-in-one; for instance, aluminum nitride AlN displays a coexistence of electrical insulating properties with high thermal conductivity [6], whereas titanium nitride TiN provides an advantageous combination of tough mechanical and thermal resistance with extremely low electrical resistivity [7]. Other nitride materials that receive considerable attention are boron nitride BN [8] and silicon nitride Si_3_N_4_ [9]. Many foreseen uses of complex nitrides, i.e., alloyed or composite, utilize the synergy of coexisting electronic and mechanical/thermal properties [1,2,3,4,10]. In this regard, one of the relatively not yet in-depth investigated composite nitride systems is made of GaN and TiN, with a potentially attractive set of combined optical, electronic, and mechanical properties.

Regarding the composite system GaN–TiN, to the best of our knowledge, no reliable thermodynamic equilibrium data on the relevant ternary element system Ti–Ga–N are available. The work on alloyed metal Ti contacts to GaN layered structures provided merely a simplified ternary phase diagram for Ti–Ga–N [11]. Moreover, there are only scarce reports on other properties of such contacts, with one study confirming Ti reacting with GaN toward TiN [12]. Some scattered reports on the room-temperature stable binary compound Ti_2_GaN confirm its crystallization as a specific layered hexagonal polytype (so-called MAX phase) [13,14,15]. In this regard, under ambient conditions, GaN can crystalize in the stable hexagonal, wurzite-type 2H form h-GaN and in the metastable cubic, sfalerite-type 3C form c-GaN or as a mixture of the two polytypes [16,17]. There are also reports on the cubic, rock salt-type polymorph rs-GaN formed under extreme solvothermal conditions, as well as, interestingly, upon stabilization by TiN/GaN multilayers [18]. Regarding TiN, it clearly tolerates some nitrogen deficiency, and it is stable as a cubic, rock salt polytype c-TiN (c-TiN_x_, x < 1). From the structural point of view, therefore, no solid solution formation is anticipated under ambient conditions between the thermodynamically stable but crystallographically different polytypes of hexagonal GaN and cubic TiN. Questions of the latter forming solid solutions with the metastable cubic GaN or significantly alloying with any of the GaN polytypes under high pressure have not yet been addressed.

We have developed throughout the years several preparation methods for nitride nanopowders such as GaN [16,19,20,21,22,23,24], AlN [20,21], and TiN [25,26], as well as for some related composites, in many cases via alternative precursor routes. Furthermore, we described for the first time the no-additive sintering of pure [27] and Mn-doped/magnetic GaN nanopowders [28] with controlled recrystallization toward mechanically robust nanoceramics. Moreover, we recently reported on no-additive HP–HT sintering of two of these nitrides binary composite systems, i.e., GaN–AlN and TiN–AlN, applying a similar precursor chemistry scheme [20,29,30]. In the system GaN–AlN [31], specifically, in addition to a proportion of the stable hexagonal polytypes of the individual nitrides, the formation of their hexagonal solid solution Al_x_Ga_1−x_N was confirmed already at the powder synthesis stage, which progressed in the sintering step to result under selected conditions in the novel nitride nanoceramics of pure Al_0.5_Ga_0.5_N. In this system, the specific reactivity of the dimeric Al- and Ga-tris(dimethylamide) precursors, {M[N(CH_3_)_2_]_3_}_2_, M = Al/Ga, Al:Ga = 1:1 (at.), mixed in hexane, was shown by solution ^1^H- and ^13^C{H}-NMR spectroscopy to have an essential impact on the nitride subsequent solid solution formation [20]. Specifically, no detectable formation of a mixed bimetallic Al/Ga-tris(dimethylamide) dimer occurred in hexane at room temperature, whereas, under reflux conditions, the mixed dimer [(CH_3_)_2_N]_2_Al-[*μ*-N(CH_3_)_2_]_2_Ga[N(CH_3_)_2_]_2_ amounted to about 50%. Such a system of intimately mixed monometallic and bimetallic dimers, when subjected to further ammonolysis reactions with ammonia, afforded a complex solid amide-imide precursor already containing –N–Al–N–Ga–N– linkages that later favored the Al_x_Ga_1−x_N solid solution formation. In the system TiN–AlN [32], no solid solution of the crystallographically different component nitrides was detected at any of the processing stages including sintering, whereas the latter stage was characteristic of the pronounced individual nitride phase segregation on the micrometer scale. This was paralleled this time by no metal-dimethylamide interactions in the hexane solution, even under reflux conditions, as confirmed by a relevant NMR study. Lastly, both systems afforded hard composite nanoceramics despite the observed microsized porosity, especially for GaN–AlN, associated mostly with facile recrystallization of GaN during sintering.

Herein, we extend the binary nitride series of GaN–AlN and TiN–AlN to include the new and unexplored system GaN–TiN through the use of the previously applied original transamination/deamination precursor chemistry via the metal imides. Equilibration of the organometallic precursors in hexane was investigated by solution NMR spectroscopy. The nitridation of the mixed metal imides was anticipated to yield synthesis-mixed GaN and TiN nanopowders, which were subjected to no-additive HP–HT sintering toward new composite GaN–TiN nanoceramics. In parallel experiments, the synthesis of GaN and TiN nanopowders was carried out, which were individually sintered to the pure nitride’s nanoceramics.

## 2. Experimental

### 2.1. Preparation of Metal–Imide Precursors from the Initial Mixed Bimetallic Tris/Tetrakis (Dimethylamide) System │{Ga[N(CH_3_)_2_]_3_}_2_/Ti[N(CH_3_)_2_]_4_│/NH_3_, Atomic Ratio Ga:Ti = 1:1 and from Individual Reference Systems of │{Ga[N(CH_3_)_2_]_3_}_2_│/NH_3_ and │Ti[N(CH_3_)_2_]_4_│/NH_3_

#### 2.1.1. Preparation of Mixed Bimetallic Imide Precursor via 3 h Reflux in Hexane Solution

Samples of {Ga[N(CH_3_)_2_]_3_}_2_ [33], 5.06 g (12.5 mmol of dimer), and Ti[N(CH_3_)_2_]_4_ [34], 5.61 g (25.0 mmol of monomer), were made according to published procedures, dissolved together in 60 mL of hexane, and refluxed under nitrogen for 3 h. In the similar but reactive Al/Ga-dimethylamide system, this was equivalent to formation of ca. 50% bimetallic dimer {Al/Ga[N(CH_3_)_2_]_3_}_2_ [20]. Upon cooling to RT and hexane evaporation, liquid NH_3_ (ca. 60 mL) was transferred onto the solid, and the suspension was stirred under ammonia reflux at ca. −33 °C for 2 h, which was followed by a 2 h NH_3_ boil-off at this temperature. The resulting white solid was evacuated at RT for 0.5 h affording the mixed polymeric Ga- and Ti-imide precursor.

#### 2.1.2. Preparation of Reference Individual Ga– and Ti–Imide Precursors

The Ga-imide [19] and Ti-imide [30] precursors for individual nitrides GaN and TiN were made, respectively, from solid gallium tris(dimethylamide) and liquid titanium tetrakis(dimethylamide) by ammonolysis of the samples in excess liquid ammonia under comparable conditions applied earlier for the mixed bimetallic imide precursor.

### 2.2. Nitridation toward Nanopowders

The mixed bimetallic imide precursor, as well as the individual precursors of Ga-imide and Ti-imide, was used in nitridation pyrolysis experiments. They were performed in a heated alumina tube under a flow of NH_3_, 0.2 L/min, for 4 h at two selected temperatures of 800 and 950 °C for the mixed bimetallic imide and Ga-imide precursors and at 800 and 1000 °C for the Ti-imide precursors, each loaded in a closed-end alumina boat. The products in the amount of ca. 1.5 g were dark brown (if containing TiN) or yellowish gray (pure GaN) free-flowing powders that were stored in a glove-box for analytical determinations. The samples for sintering experiments were loaded in glass ampoules that were sealed under vacuum and opened directly before sintering.

### 2.3. High-Pressure and High-Temperature Sintering

Upon ampoule opening, the powders were removed and briefly handled in air prior to high-pressure and high-temperature sintering, as described earlier [27,28,31,32]. Specifically, the powders were sintered for 3 min in a high-pressure torroid cell at 650, 1000, and 1200 °C under pressure of 7.7 GPa, yielding dark brown/golden or gray ceramic pellets, D = 4 mm, thickness ca. 2–4 mm. For Vicker’s hardness determinations on a pellet, one of its sides was polished. For other measurements, the pellets were ground in an agate mortar and used as such.

**Figure 1:**
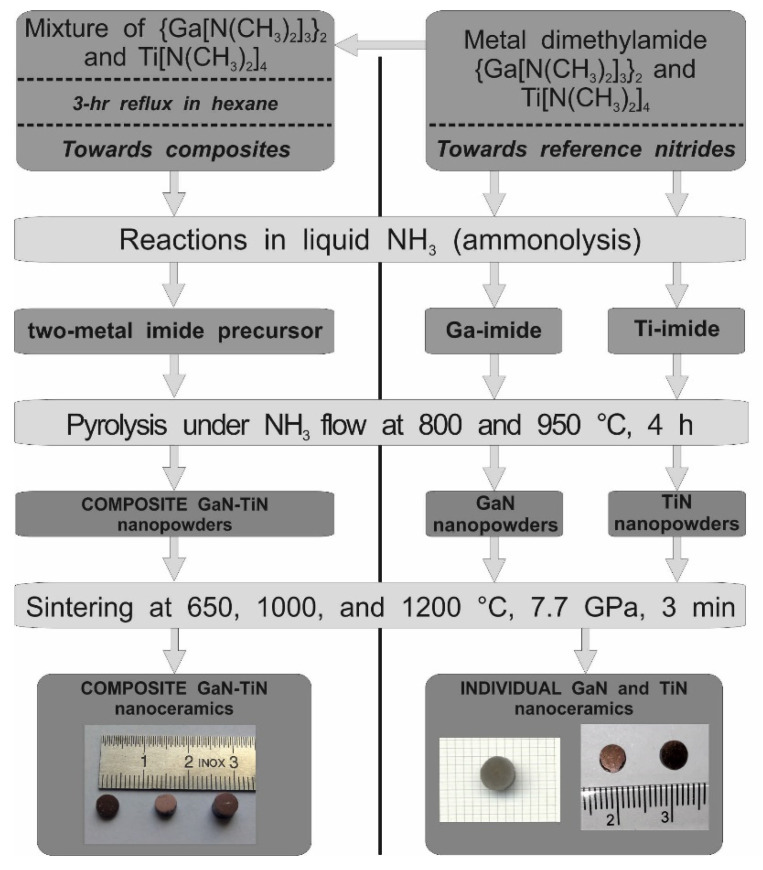
Sequence of experimental steps in anaerobic preparation and subsequent high-pressure and high-temperature sintering of composite and individual nanopowders in the system GaN–TiN. Snapshots of typical nanoceramics are shown at the bottom: left—brown composite pellets; right—gray GaN pellet and golden-brown TiN pellets.

The major steps in the synthesis and sintering of the nitride materials are shown in Figure 1.

### 2.4. Powder and Nanoceramic Sample Labeling

The nitride nanopowders prepared from the bimetallic imide precursor were labeled as Composites with an additional reference to nitridation temperature. For example, after the nitridation at 800 and 950 °C, two products were obtained, i.e., Composite_800 and Composite_950, respectively. The nitride nanopowders made from pure metal imide precursors were the individual nitrides of GaN and TiN, which were labeled accordingly. For example, for Ga-imide pyrolyzed at 800 and 950 °C, the two powder products were GaN_800 and GaN_950, whereas, for Ti-imide pyrolyzed at 800 and 1000 °C, the resulting products were TiN_800 and TiN_1000, respectively. The sintered ceramics had names of the related nitride powders with suitable postfixes for sintering temperature, e.g., Composite_800_sint_650, GaN_800_sint_1000, or TiN_1000_sint_1200.

### 2.5. Characterization

^1^H- and ^13^C{H}-NMR spectra were recorded using a Bruker Avance III 600 MHz spectrometer (Bruker, Billerica, MA, USA) at 300 K equipped with the nitrogen cryo-probe head. Spectra were recorded in C_6_D_6_ solutions contained in sealed 5 mm glass tubes, and the ^1^H and ^13^C chemical shifts were referenced to the residual solvent signals with TMS set to zero ppm. The spectra for the mixed bimetallic dimethyl amides were acquired shortly after the 3 h hexane reflux equilibration. Powder XRD determinations were done for all nitride products (powders and sintered ceramics) by Empyrean PANalytical (Malvern, UK), Cu Kα source, 2 Θ = 20–80°. Due to very small crystallite sizes, the peaks were broadened and frequently overlapped, resulting in decreased accuracies of crystallite cell parameter determinations down to ca. 0.01 Å. XRD-derived average crystallite sizes were evaluated from Scherrer’s equation, applying the Rietveld refinement method. SEM/EDX imaging and analytical data were acquired for carbon-coated samples with a Hitachi Model S-4700 scanning electron microscope (Hitachi, Tokyo, Japan). Raman spectroscopy was done with a WITec Alpha 300M+ spectrometer (WITec, Ulm, Germany) equipped with Zeiss optics (50×). Measurements were carried out using a 488 nm diode laser. Four accumulation of 30 s scans were gathered at each point. Baseline subtraction was performed using WITec’s software (ProjectFive Plus, WITec, Ulm, Germany). Helium densities were determined using a Micromeritics AccuPyc 1340 pycnometer (Micromeritics, Norcross, GA, USA). The Vicker’s hardness (H_v_) tests were performed on microhardness tester FutureTech FM-700 (Future-Tech Corp., Fujisaki, Japan) with a 100 and 300 gf (gram-force) load on a polished pellet surface, 10 s, and hardness was expressed in GPa. The datasets for the two loads were comparable, and the more consistent set of H_v_ values, recorded for 300 gf, was selected for discussion. Typically, 5–10 measurements were carried out for a pellet to calculate an average H_v_ value and its standard deviation.

## 3. Results and Discussion

### 3.1. Powder Preparation via Transamination/Deamination/Nitridation Chemistry

The driving force behind facile preparation of the metal imides as direct precursors for nitride nanopowders is an efficient transamination in refluxing ammonia of both individual Ga(III)- and Ti(IV)-dimethylamides, which is followed at room temperature by spontaneous multistep deamination in the solid state to approach the theoretical metal imide formulae of {Ga(NH)_3/2_} and {Ti(NH)_2_}. The resulting polymeric Ga-imide precursor is then subjected to nitridation pyrolysis at increased temperatures, preferentially under an ammonia flow to complete the deamination steps according to {Ga(NH)_3/2_} → GaN + 1/2 NH_3_ and to form the nitride nanopowders with no separation efforts. For the theoretical Ti-imide Ti(NH)_2_, the deamination is accompanied by redox reactions as approximated by {Ti(IV)(NH)_2_} → Ti(III)N + 1/2 NH_3_ + 1/4 H_2_ + 1/4 N_2_. The general transamination/deamination chemistry was precedented in the 1980s [29,30] and, specifically, it was detailed by us for the dimethylamide derivatives of gallium [19,20], manganese-doped gallium [28], aluminum [20], mixtures of gallium and aluminum [20,31], and mixtures of aluminum and titanium [32]. It was also demonstrated to work for some trimethylsilylamides as reported for the preparation of manganese nitride *η*-Mn_3_N_2_ from Mn-bis(trimethylsilyl)amide [35].

It is instructive to recall that, in the dimeric Al-tris(dimethylamide) plus dimeric Ga-tris(dimethylamide) system refluxed in hexane, the presence of some mixed-metal dimeric Al/Ga-tris(dimethylamide) was confirmed in the equilibrated solution and shown to be linked to the formation of known Al_x_Ga_1−x_N alloy upon further nitridation workup [20,31]. In this regard, the course of transamination/deamination reactions in such a bimetallic dimethylamide system is thought to yield some mixed M-amide-imide species (M = Al/Ga) and, eventually, to facilitate metal nitride solid solution formation as indeed observed. In the actual bimetallic system of dimeric Ga-tris(dimethylamide) and monomeric Ti-tetrakis(dimethylamide), the formation of the mixed bimetallic Ga/Ti-tris/tetrakis/(dimethylamide) seems unlikely due to steric factors, although the possibility cannot be excluded. In the recently reported by us and very closely related bimetallic system of dimeric Al-tris(dimethylamide) and monomeric Ti-tetrakis(dimethylamide), no equilibration in hexane solution toward mixed bimetallic molecular species was observed, which was reflected by no solid solution formation in that system made at the end of the individual nitrides of cubic TiN and hexagonal AlN [32].

**Figure 2:**
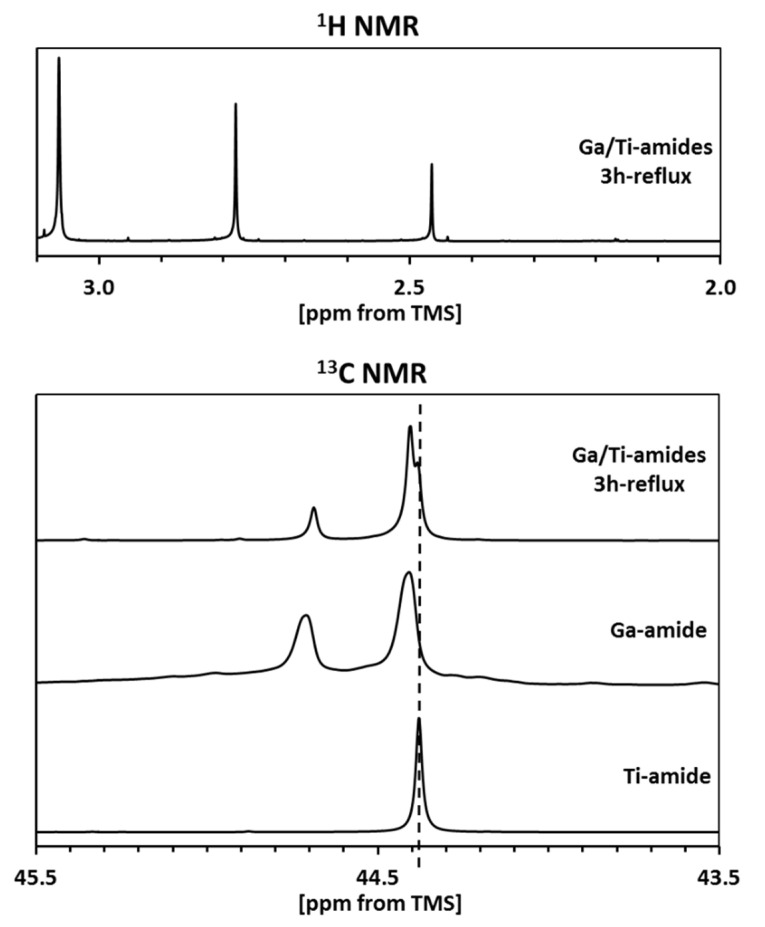
^1^H- (upper) and ^13^C- (lower) NMR spectra in C_6_D_6_ of bimetallic M-dimethylamide mixture (M = Ga, Ti; Ga/Ti = 1/1 at.) refluxed in hexane. ^1^H-NMR spectrum is shown for bimetallic product only, whereas ^13^C-NMR results also include spectra for individual/pure M-dimethylamides. The dashed line shows the position of ^13^C-NMR resonance for monomeric Ti-dimethylamide and serves as a guide for the eye only.

In order to check the possibility of the molecular metal-mixed Ga/Ti-dimethylamide species formation, the ^1^H- and ^13^C-NMR spectra were collected for a solid sample isolated after the equilibration step in refluxing hexane (Figure 2).

The ^13^C-NMR spectrum for pure Ti-tetrakis(dimethylamide) (Figure 2, bottom) is consistent with its monomeric character, resulting in the single signal at 44.38 ppm [32]. On the other hand, the spectrum for pure Ga-tris(dimethylamide) confirms its dimeric character in the solution with four terminal and two bridging –N(CH_3_)_2_ groups to yield two nonequivalent carbon sites at 44.40 and 44.68 ppm with the intensity ratio 2:1 [20]. For the equilibrated mixture (Figure 2, top parts), the carbon spectrum is essentially unchanged upon superimposing the two spectra for the pure M-dimethylamides. The small variations in peak positions on the order of 0.01 ppm are likely due to concentration-dependent effects and are insignificant from the point of view of major structural changes. All this is consistent with the proton spectrum for the refluxed product (Figure 2, top), which shows resonances expected for the individual M-dimethylamides in the mixture—a single peak at 3.08 ppm for the monomeric Ti component [34] and two peaks at 2.79 and 2.48 ppm with 2:1 intensity ratio, respectively, for the dimeric Ga component with four terminal and two bridging –N(CH_3_)_2_ groups [20]. It appears that, on the NMR time scale, the amide mixture is just made of the dimeric Ga-tris(dimethylamide) and monomeric Ti-tetrakis(dimethylamide) with no essential bimetallic amide formation. Therefore, this stage of precursor processing is expected to be indifferent for a potential and otherwise unlikely formation of the metastable bimetallic Ti_x_Ga_1−x_N along the nitridation pathways to composite nitride nanopowders.

**Figure 3:**
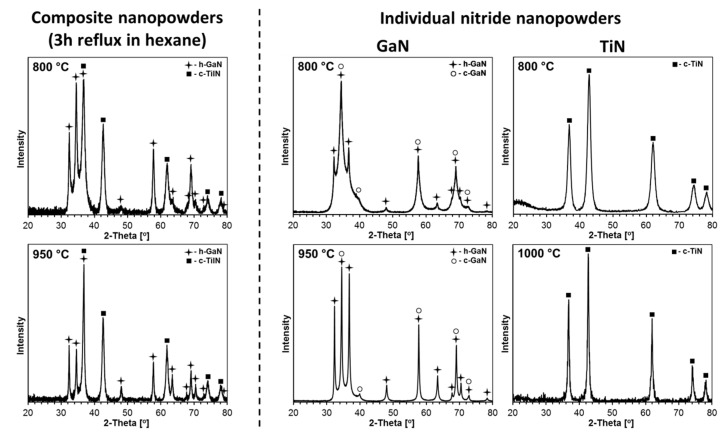
XRD patterns of composite (**left**) and individual (**right**) nitride nanopowders prepared at 800 and 950 °C in the system GaN–TiN. Note that the pattern for TiN nitrided at 1000 °C is shown instead of the common reference 950 °C.

The XRD patterns for the nanopowders from two composite nitrides prepared at 800 and 950 °C and reference pure nitrides, GaN, prepared at 800 and 950 °C, as well as TiN, prepared at 800 and 1100 °C, are displayed in Figure 3. The calculated cell parameters for the determined GaN and TiN polytypes in composites and in individual nitrides, as well as their proportions and estimated average crystallite sizes, are included in Table 1.

The composite nanopowders from both nitridation temperatures are 1:1 mixtures of the thermodynamically stable hexagonal GaN and cubic TiN with no evidence for any metastable bimetallic nitride. Interestingly, the composites contain exclusively h-GaN, and there are no detectable quantities of the c-GaN polytype that is otherwise present in significant proportions in the reference pure GaN both from 800 °C (h-GaN/c-GaN = ca. 1/1) and 950 °C (h-GaN/c-GaN = ca. 2/1), as seen in Table 1. In this regard, the anaerobic method used for GaN preparation often specifically yields a mixture of h-GaN and c-GaN, especially, at relatively low nitridation temperatures. A higher temperature results in lower amounts of the metastable c-GaN due to its conversion to the stable h-GaN. It is apparent that the nitridation of the two metal-imide precursors (mixture of Ga-imide and Ti-imide) prevents c-GaN formation in the resulting composites. If present, c-GaN could possibly participate in making some solid solutions c-Ti_x_Ga_1−x_N with the crystallographically alike polytype of c-TiN. The low likelihood for such a solid solution formation stems mainly from a relatively large mismatch between the recorded lattice parameters of c-GaN (a = 4.50–4.51 Å) and c-TiN (a = 4.24–4.25 Å). The observed *a*-constants of 4.24 and 4.25 Å for the c-TiN component in the composites are very close to the reported typical values of 4.23–4.24 Å for pure c-TiN [36,37,38] and the value of 4.24 Å found here for the reference pure c-TiN from both nitridation temperatures. Some doping of the c-TiN lattice with gallium centers cannot be excluded, however, given the two-metal-precursor system mixed on the molecular level, thus undergoing conversions to the nitrides.

The application of the higher nitridation temperature of 950 °C for the composite system resulted in visibly increased average crystallite sizes of both components compared with 800 °C, i.e., D_av_ for h-GaN increased from 27 to 55 nm and, for c-TiN, it increased from 10 to 14 nm. It is clear that the size effect is much more pronounced for the h-GaN component that, remarkably, reaches its stability limits in the range 950–1000 °C [22]. These values can be confronted with data for the individually prepared pure gallium nitride and titanium nitride nanopowders. As discussed earlier, the former nitride is present as a mixture of two polytypes confirmed at both nitridation temperatures. In this regard, for the nitridation temperatures of 800 and 950 °C, the h-GaN component in pure gallium nitride in relation to the composites with D_av_’s of 27 and 55 nm shows comparable D_av_’s of 17 and 50 nm, respectively. Similarly, the 800 °C prepared pure c-TiN with D_av_ of 8 nm compares satisfactorily with the D_av_ of 10 nm for the c-TiN component in the related composite. There is a lack of XRD data for c-TiN from the 950 °C nitridation, but the available data for the very closely related 1000 °C prepared c-TiN qualitatively confirm an anticipated D_av_ increase to reach 19 nm on application of the higher nitridation temperature. This particular piece of data can find relevant use when discussing the effects of sintering temperatures in the 650–1200 °C range, including the 1000 °C level, on crystallite growth of the components (vide infra).

### 3.2. High-Pressure and High-Temperature (HP–HT) Powder Sintering

The as-synthesized powders were sintered with no additives following the procedure previously reported by us for nanopowders in the related systems of AlN–GaN [31] and TiN–AlN [32]. These are quite relevant cases since analogous precursor routes and powder nitridation schemes were employed. Specifics are concerned with some differences in nitridation and sintering temperatures. Specifically, in this study the powder nitridation temperatures were 800 and 950 °C (except for 800 and 1000 °C used in the case of pure TiN), and sintering temperatures for the composite powders were 650, 1000, and 1200 °C to be compared with the most relevant case of the system AlN–GaN with the same nitridation levels at 800 and 950 °C and sintering at 650 and 1000 °C, respectively. The lower nitridation temperature of 800 °C was used in both cases to afford powders with average crystallite diameters of several nanometers. The higher temperature of 950 °C provided larger crystallite diameters, whereas its level was dictated by an increased thermal instability of nanocrystalline GaN above it [22]. In this regard, the TiN component in the current system of GaN-–TiN is reasonably stable up to at least 1200 °C [25]. The selection of sintering temperatures was based on the same two criteria in all these studies. First, the lower temperature of 650 °C was below the lower nitridation temperature of 800 °C and, therefore, sintering was anticipated to occur with no temperature-induced recrystallization. Second, the higher temperatures of 1000 and 1200 °C were above the higher nitridation temperature of 950 °C with possible positive impact on crystal growth and, consequently, sintering with temperature-induced recrystallization. Additionally, the application of the 1200 °C sintering temperature for a short sintering time of 3 min and under the pressure of 7.7 GPa, i.e., a relatively high temperature regarding the thermal stability of GaN nanopowders (vide intra), was intended to check any interactions of decomposing GaN with the stable TiN component under such conditions. We also expected to observe similar effects of high pressure as previously that at the lowest sintering temperature and no crystal growth caused a net nanocrystallite “crushing”/lowering of average crystallite sizes, whereas, at the higher sintering temperature and associated temperature-induced crystal growth, the “crushing” competed with crystal growth. This serves to recall that high pressures are mostly used to significantly speed up sintering of powders. Lastly, the HP–HT sintering resulted in hard round-shaped pellets made of composite or individual nitride nanoceramics, as shown in Figure 1. The pellets containing TiN were brown or golden brown, while those of pure GaN were gray.

**Figure 4:**
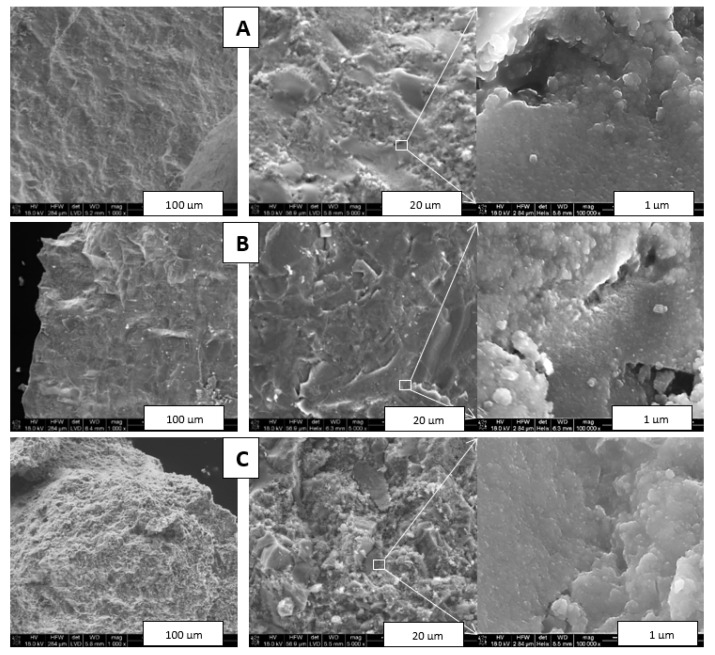
SEM images of intentionally fractured nanoceramics sintered from Composite_950: (**A**)—Composite_950_sint_650, (**B**)—Composite_950_sint_1000, (**C**)—Composite_950_sint_1200. Areas in white-edged rectangles are magnified by a factor of 20 and are shown in adjoining images.

**Figure 5:**
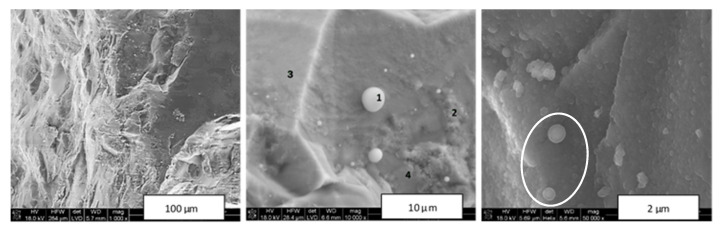
SEM images of intentionally fractured nanoceramics of Composite_800_sint_1200. Numbers in the middle image show spots subjected to EDX analysis (see, text). In the right image, apparently partially molten shell–core spheres are encircled by white ellipse.

Figure 4 shows typical images of intentionally fractured fragments of composite nitride nanoceramics made from Composite_950 at all three sintering temperatures, i.e., 650, 1000, and 1200 °C, while Figure 5 presents similar graphics for nanoceramics made from Composite_800 sintered at 1200 °C.

The fractures in Figure 4 confirmed a dense material packing of homogeneous appearance at low magnification (left row). At higher magnification, the characteristic feature of all composite ceramics was the presence of intermixed and relatively large (up to several tens of micrometer) two distinct domains each with specific appearance, i.e., solid, seemingly dense/homogeneous islands with smoothly fractured surfaces that were embedded in an apparently porous matrix with irregularly shaped surfaces (middle row). The interfaces between the two types of domains were easily discerned. The EDX analysis confirmed that the solid areas were prevailingly TiN, whereas the surface-irregular matrix was significantly enriched in GaN. Furthermore, quite numerous micron- and submicron-sized pores could be seen, especially at the highest magnifications (right row). These images confirmed also the homogeneous, grainy in appearance submicron morphology that, upon inspection, was made of similar in size and densely agglomerated/sintered nanocrystallites in the several tens of nanometer range. At the higher magnifications for the 1200 °C ceramics, there were occasionally seen some regularly shaped relatively large crystallites in a few micrometer size range (Figure 4C). Their characteristics were consistent with hexagonal GaN, and their size could be linked to a rapid crystal growth competing with decomposition under the sintering conditions. It should be noted, however, that no metallic Ga was observed in any of the ceramics examined in Figure 4. In conclusion, the HP–HT sintering of the Composite_950 nanopowder appeared to be associated with significant phase separation and resulted in the intermixed nitride-specific domains on the several micrometer scale. This is exactly what was observed in the related system of TiN–AlN [32], which supports a similar sintering mechanism in both composite nitride systems.

All the above observations applied closely to similar sintering of the Composite_800 nanopowder. In this case, however, an SEM examination of the Composite_800_sint_1200 nanoceramics, sintered at the highest applied temperature of 1200 °C, provided evidence for partial decomposition of the GaN component and formation of metallic gallium (Figure 5). In a few areas, micron- and submicron-sized droplets were seen (Figure 5, middle). The EDX analysis carried out for the middle image confirmed the droplets being elemental Ga (point 1), with the solid-looking areas adjoining to pores being significantly enriched in GaN to pure GaN (point 4), while the other dense areas were enriched in TiN (points 2 and 3). This is consistent with our previous observations that the applied sintering temperatures above 1000 °C, here 1200 °C, may cause not only decomposition of some GaN, but also concomitant rapid GaN crystal growth, making the initial GaN domains (agglomerates of nanocrystallites) appear dense (aggregates of microcrystallites) while also showing evolving porosity due to excessive mass transfer during such recrystallization. At the highest magnifications that resolved nanosized objects, shell droplets a few hundred nanometers in size, as if split-broken, attached to a homogeneous grainy/nanosized solid bulk could occasionally be observed (Figure 5, right). The overall round-shaped appearance of the objects and the presence of the shells suggested a partial surface decomposition of small GaN agglomerates with formation of a layer of initially molten Ga (m.p. 29.8 °C) on the core made of not yet decomposed crystallites of GaN.

In concluding this section, there clearly must be a strong enough driving force during HP–HT sintering for nitride segregation and extended agglomeration via particle displacement even without recrystallization and associated mass transport phenomena. This takes place even for the 650 °C sintering that is neutral to temperature-induced crystal growth. Since the precursor nanopowders were synthesis-mixed on a submicron-size scale, it is the application of high pressure which appears to promote the alike nitride particle agglomeration and result in the different nitride phase segregation on a few micron scale. It is quite likely that, under such circumstances, the Van der Waals forces among the chemically and crystallographically similar nitride particles play an essential role in this phenomenon.

**Figure 6:**
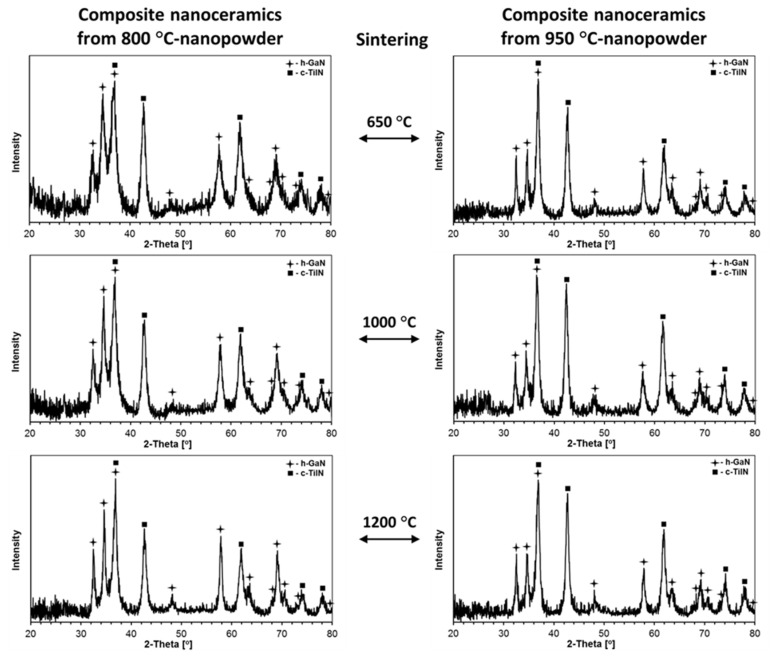
XRD patterns of composite nitride nanoceramics sintered from nanopowders of Composite_800 (**left**) and Composite_950 (**right**) at 650, 1000, and 1200 °C, 7.7 GPa, 3 min.

**Figure 7:**
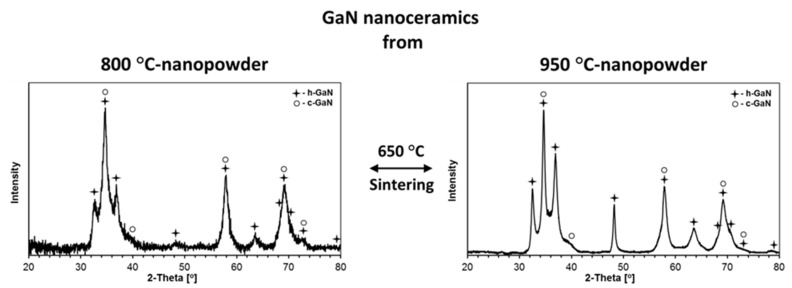
XRD patterns of pure GaN nanoceramics sintered from nanopowders of GaN_800 (**left**) and GaN_950 (**right**) at 650 °C, 7.7 GPa, 3 min.

The powder XRD patterns for all ceramics produced from the composite nanopowders are shown in Figure 6 and, for reference purposes, the XRD patterns for selected ceramics from pure GaN are displayed in Figure 7. The evaluated lattice cell constants and average crystallite sizes are included in Table 2.

It can be noted that all composite nanoceramics were made of the stable hexagonal GaN and cubic TiN polytypes with no detectable amounts of the metastable cubic GaN that was present in varying quantities in the starting powders. This is consistent with the conversion of all c-GaN to h-GaN upon the HP–HT sintering conditions. As observed by us earlier in the systems AlN–GaN [31] and TiN–AlN [32], the sintering temperature of 650 °C, lower than both powder nitridation temperatures (800 and 950 °C), yielded mostly smaller average crystallite sizes D_av_’s in such nanoceramics compared with the starting powders. This can be illustrated by comparing the D_av_’s (h-GaN/c-TiN) for the Composite_800 and Composite_950 nanopowders (Table 1) with their 650 °C derived nanoceramics (Table 2). For the composite nanopowders, the D_av_’s (h-GaN/c-TiN) were 27 nm/10 nm for 800 °C and 55 nm/14 nm for 950 °C, whereas, for the 650 °C composite-derived nanoceramics, they were, respectively, 19 nm/10 nm and 35 nm/12 nm. As previously, we assigned such an outcome to “crushing” of the nanocrystallites by the extremely high pressure in the absence of crystal growth, here more prominent for the larger-sized h-GaN component. However, at the sintering temperatures of 1000 and 1200 °C (sintering with recrystallization), a competition between “crushing” and temperature-induced crystal growth yielded different results. In particular, for the 1000 °C composite-derived nanoceramics, the D_av_’s were found for the respective starting nanopowders at 20 nm/12 nm and 33 nm/13 nm, while, for the 1200 °C derived nanoceramics, the average particle sizes for the polytypes were, respectively, 26 nm/13 nm and 37 nm/14 nm. It is interesting to note that, at the discussed sintering temperatures, the recrystallization was more pronounced for h-GaN than for c-TiN, and was still confined to the crystallite low nanosize range.

It is instructive to relate some of these structural features of the composite nanoceramics to the individual nitride nanoceramics at comparable HP–HT sintering conditions (see Table 1 and Table 2). When considering the case of pure GaN, sintering at 650 °C of both 800 and 950 °C nanopowders resulted in nanoceramics with relatively increased proportions of the stable h-GaN polytype relative to c-GaN, and sintering at 1000 °C yielded in both cases only the stable h-GaN. This serves to recall that sintering of the composite nanopowders resulted exclusively in the formation of the h-GaN component. For the sintering at 650 °C, the D_av_’s for h-GaN were comparable with the values in the related 650 °C composites, whereas the sintering of pure GaN with recrystallization at 1000 °C yielded D_av_ = 160 nm for GaN_800 and D_av_ = 31 nm for GaN_950. Apparently, the temperature spread between the powder preparation and powder sintering temperatures, which was much higher for the GaN_800 precursor, i.e., 200 vs. 50 °C, is a major driving force behind the rate of crystal growth. It is also clear that sintering with recrystallization at 1000 °C created much less restrained conditions for crystal growth in pure GaN nanopowders than in the composite nanopowders, the latter being consistent with a TiN-component thinning effect. This is supported by the h-GaN data with D_av_ = 160 nm for GaN_800_sint_1000 vs. D_av_ = 20 nm for Composite_800_1000 with the 200 °C spread, while there was apparently no such effect for the 50 °C spread, yielding D_av_ = 31 nm for GaN_950_1000 vs. D_av_ = 33 nm for Composite_950_1000. There was no sintering of pure GaN done at 1200 °C in this study due to thermal instability of the pure nitride [22]. However, as revealed earlier by the SEM study, the 1200 °C sintering of the composite powders was successfully accomplished with only traces of GaN decomposition, which we attributed to the action of high pressure and the relatively short sintering time of 3 min. As far as the case of pure TiN is concerned, some related data were already published by us for the TiN–AlN system [32], i.e., in the case where the powder nitridation temperatures were 800 and 1100 °C and the sintering ones were 650 and 1200 °C. The closely related case was that of the TiN_800 nanopowder (a = 4.24 Å, D_av_ = 8 nm) sintered at 650 °C to yield the TiN_800_sint_650 nanoceramics (a = 4.24 Å, D_av_ = 8 nm), consistent with no essential changes in structure and particle size parameters, including no crystallite “crushing”, in such sintering without recrystallization. Sintering at 1200 °C resulted in merely moderate recrystallization for TiN_800_sint_1200 (a = 4.24, D_av_ = 13 nm). The data for pure TiN can now be referred to the relevant cases of the GaN–TiN composites in this study. For Composite_800_sint_650, the D_av_ for the c-TiN component equal to 10 nm can be favorably compared to 8 nm in pure TiN_800_sint_650. Similarly, for Composite 800_sint_1200, the relevant D_av_ of 10 nm can be compared with 13 nm for pure TiN_800_sint_1200 (quoted in [32]). It is apparent that the growth of TiN was somewhat restrained in the composites; however, as mentioned before, the changes in D_av_’s for TiN were clearly smaller than found for GaN in all comparable cases.

**Figure 8:**
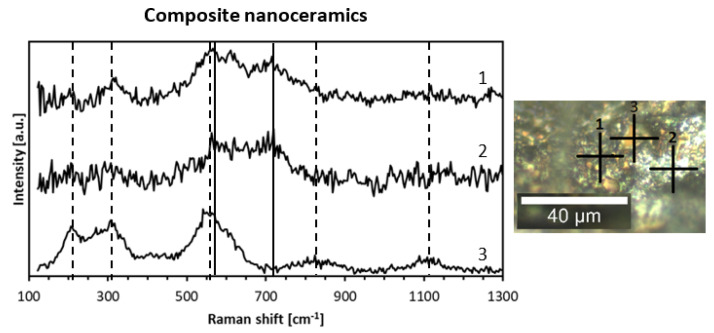
Raman spectra (**left**) for nanoceramics of Composite_800_sint_1000 at points 1–3 (**right**). Vertical lines are shift positions for various modes of TiN (dashed) and GaN (solid) and serve as guides for the eye, only.

**Figure 9:**
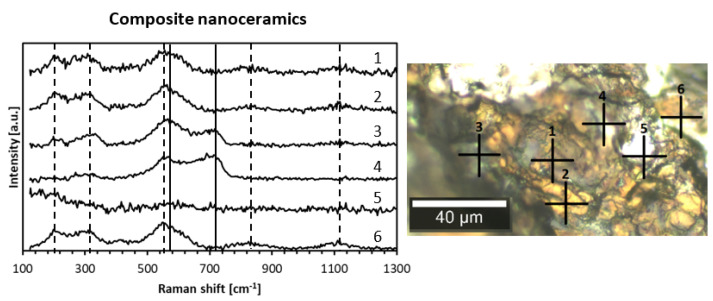
Raman spectra (**left**) for nanoceramics of Composite_950_sint_1000 at points 1–6 (**right**). Vertical lines are shift positions for various modes of TiN (dashed) and GaN (solid) and serve as guides for the eye, only.

Raman spectroscopy is a versatile tool to probe composite nanoceramics from the point of view of phase domains. In this regard, the first-order Raman spectra are forbidden for perfect O_h_ symmetry in cubic TiN. Due to high propensity of the nitride for non-stoichiometry with nitrogen deficiency (as in TiN_x_, x < 1) with the then active modes, Raman spectroscopy is often used for various titanium nitride products such as single crystals [39], thin layers [40], and powders [41]. As examples, Figure 8 shows the Raman spectra for three points (indicated as crosses in the adjoining microscopic image) for the nanoceramics of Composite 800_sint_1000, and Figure 9 includes such spectra for six points for Composite_950_sint_1000.

Raman confocal microscopy for the nanoceramics confirmed the distinct micrometer-sized domains discussed earlier during SEM analysis, i.e., solid looking and quite regularly shaped golden-brown areas (TiN) embodied in the bright porous-type matrix (GaN). Typical features of the Raman spectrum for a few micrometer-sized TiN domain labeled 3 is displayed in Figure 8, curve 3. The spectrum consisted of square-like shaped acoustic phonons with TA and LA modes at ca. 220 cm^−1^ to 320 cm^−1^, triangular-like shaped TO modes at 560 cm^−1^ with a weak contribution of the LO modes up to 620 cm^−1^, and two wide maxima of overtones at ca. 820 (LA + TO) and 1120 cm^−1^ (2 TO), as reported in the literature for nearly stoichiometric titanium nitride [42]. The square and triangular-like features resulted from defect-induced first-order Raman scattering in sub-stoichiometric TiN_x_ (x < 1). The appearance of the second-order overtones was consistent with a relatively small N-deficiency in the titanium nitride domains since the intensities of the second-order signals are reported to decrease rapidly with increased non-stoichiometry [43]. The Raman spectra for the surface-uneven and apparently porous GaN domains labeled 1 and 2 are presented in Figure 8, curves 1 and 2. Since the most intense band E_2_ (high) for h-GaN showed up at ca. 570–580 cm^−1^ [21,44], it can be easily confused with the strong TO mode at 560 cm^−1^ for TiN. However, the appearance of an active band at ca. 720–730 cm^−1^ was diagnostic for GaN being in the range of the A_1_ (LO) mode [21], especially when coupled with the absence of the square-like shaped acoustic phonons (TA and LA modes) typical for TiN. Eventually, for Figure 8 and Composite_800_sint_1000, spectra 1 and 2 were assigned to GaN and spectrum 3 was assigned to TiN, confirming nitride separation on the micrometer scale in these nanoceramics. Such a conclusion is fully corroborated by the Raman spectra for Composite_950_sint_1000 shown in Figure 9. Curves 1, 2, and 6 were consistent with TiN domains, curve 4 was consistent with GaN domains, and curve 3 represented both phases with prevailing GaN. Interestingly, curve 5 for a very bright point 5 showed no distinct spectral features. It was tempting to assign this to metallic gallium due to a likely beginning of GaN decomposition at the sintering temperature of 1000 °C. In this regard, this serves to recall that droplets of metallic Ga were seen by SEM after sintering of one of the composites at a higher temperature of 1200 °C.

The Vicker’s hardness and helium density data for the nanoceramics are compiled in Table 3. The Vicker’s hardness, H_v_, was measured by an indentation method under two loads of 100 and 300 gf (gram-force), yielding comparable results, and the data-set for 300 gf is used for discussion. In this regard, too low load (below ca. 200 gf) indents often displayed a dependence of H_v_ on indent depth, known as the indentation size effect [45]. The H_v_ values for the sintered pellets in the current system GaN–TiN were relatively moderate to high in the range 7.5 to 13.2 GPa. The lowest value of 7.5 GPa was determined for nanoceramics of Composite_950_sint_650 that were sintered without recrystallization to be compared to the highest of 13.2 GPa for two nanoceramics sintered with recrystallization, namely, the nanoceramics of Composite_800_sint_1200 and Composite_950_sint_1000. Interestingly, in the series of three nanoceramics made from Composite_800, when compared to sintering at 1000 °C, there was a noticeable increase in H_v_ upon sintering at 1200 °C, whereas, for Composite_950, there was an equally noticeable decrease in H_v_ for this temperature. In the latter case, this coincided with SEM-detected GaN decomposition with metallic Ga formation, which could have been responsible for loosening interparticle binding with deterioration of the hardness. Given the established impact in HP–HT sintering at high pressure (nanocrystallite “crushing”, speeding up compaction) and high temperature (sintering without or with recrystallization, rates of diffusion/recrystallization and pore formation, possible GaN decomposition), the weighted-out interplay of these two phenomena appeared to determine the final hardness. It is clear, however, that, within the component stability regimes, the recrystallization/crystal growth conditions were crucial for material hardness, while some accompanied microsized porosity formation did not prevent the formation of relatively hard nanoceramics in the system. The H_v_ values in Table 3 can be compared to the individually sintered nitrides. In this regard, the relevant pure h-GaN nanoceramics made by us from GaN_800_sint_650 and GaN_800_sint_1000 showed H_v_’s of 13.6/15.0 and 10.0 GPa, respectively, whereas those made from GaN_950_sint_650 and GaN_950_sint_1000 showed, respectively, H_v_’s of 10.6 and 17.4 GPa [28]. It is interesting to note that the H_v_ values for the GaN nanoceramics were in most cases larger than the reference literature value of 11 GPa [46]. At the same time, notably high H_v_‘s of 15.5 and 19.7 GPa were recorded by us for pure c-TiN nanoceramics from TiN_800_sint_650 and TiN_800_sint_1200, respectively [32], and H_v_‘s of 9.2–15 GPa for few micrometer thick layers of TiN were shown by others [47]. If one relates the Vicker’s hardness of the composite nanoceramics in this study to the hardness of the individual nitride nanoceramics, generally, slightly higher hardness was found for the latter, and the hardness of the significantly recrystallized and visibly porous h-GaN component appeared to be a limiting factor in the former.

The helium density data, which were the real/skeletal densities while possibly supporting (or not) the occurrence of closed pores, provided further insight into microstructure of the nanoceramics (Table 3). The densities for the pool of the sintered composites ranging from 4.25–4.66 g/cm^3^ were referred to 5.73 g/cm^3^, i.e., a theoretical value calculated for a 1:1 mixture (on the molar basis) of h-GaN (6.15 g/cm^3^) and c-TiN (5.24 g/cm^3^). The measured densities in this study were 74–81% of this value (Table 3, % theor.). In our previously reported data on the TiN–AlN system [32], the related densities were found in a very similar range of 70–82%, supporting a similar closed porosity evolution upon HP–HT sintering in both systems. It is interesting to relate these densities to those determined for the sintered pure nitrides, i.e., ranges of 85–92% for GaN [31] and 84–90% for TiN [32], and the comparison points to visibly lower densities determined for the GaN–TiN composites. This serves to recall that the SEM examination of the nanoceramics supports a quite extensive microporosity evolution associated mostly with the GaN domains. The relatively low densities of the composites, similarly to the case of the TiN–AlN system, appear to result from the complex nitride segregation–crystal growth–pore formation processes that are kinetically frozen and consolidated due to the short time and diverse mass transport rates for the GaN and TiN components during the HP–HT sintering of their reaction-mixed nanopowder composites.

## 4. Conclusions

The in situ synthesis-mixed nanopowders of h-GaN and c-TiN, prepared via the original chemistry carried out for solution-mixed precursors, constituted an advantageous system for high-pressure (7.7 GPa) and high-temperature (650–1200 °C) sintering with no additives toward composite nitride nanoceramics. The application of different nitridation temperatures of 800 and 950 °C enabled control over the average crystallite size of each of the powder components and established the initial conditions for sintering with or without recrystallization. Sintering without recrystallization at 650 °C, i.e., below both powder nitridation temperatures, resulted in hard nanoceramics that displayed a crystallite size “crushing” effect due to the applied very high pressure. Sintering with recrystallization at 1000 and 1200 °C, i.e., above the powder nitridation temperatures, yielded equally hard or better nanoceramics with increased average crystallite sizes of both components. No solid solution formation of the nitrides was observed in the powder preparation and/or sintering stages. An important observation was the progression on sintering of phase separation of the synthesis-mixed GaN and TiN nitrides in the nanopowders toward the formation of their distinct micrometer-size domains, some with microsized porosity.

## Figures and Tables

**Table 1:** XRD-derived lattice parameters and average crystallite sizes determined for composite and individual nitride nanopowders prepared at 800 and 950 °C in the system GaN–TiN with Ga:Ti = 1:1 molar ratio.

Composite Nanopowders	Nitridation		Pure GaN	Nitridation		Pure TiN	Nitridation	
	800 °C	950 °C		800 °C	950 °C		800 °C	1000 °C
h-GaN:			h-GaN:					
(mol.%)	(50)	(50)	(mol.%)	(50)	(70)			
a (Å)	3.20	3.19	a (Å)	3.19	3.19	n/a	n/a	n/a
c (Å)	5.21	5.18	c (Å)	5.20	5.19			
D_av_ (nm)	27	55	D_av_ (nm)	17	50			
c-TiN:			c-GaN:			c-TiN:		
(mol.%)	(50)	(50)	(mol.%)	(50)	(30)	(mol.%)	(100)	(100)
a (Å)	4.25	4.24	a (Å)	4.51	4.50	a (Å)	4.24	4.24
D_av_ (nm)	10	14	D_av_ (nm)	5	10	D_av_ (nm)	8	19

**Table 2:** XRD-derived crystallographic cell parameters *a* and *c* and average crystallite sizes D_av_’s determined for composite nanoceramics in the system GaN–TiN sintered at 650, 1000, and 1200 °C and pure GaN nanoceramics sintered at 650 and 1000 °C. Note that relevant cases of individual TiN nanoceramics are included in [32].

Composite Nanoceramics	Sintering			Individual Nanoceramics	Sintering	
from Nanopowder	650 °C	1000 °C	1200 °C	from Nanopowder	650 °C	1000 °C
**Composite_800 °C**				**GaN_800 °C**		
h-GaN:				h-GaN:		
(mol.%)	(50)	(50)	(50)	(mol.%)	(70)	(100)
a (Å)	3.20	3.20	3.19	a (Å)	3.18	3.19
c (Å)	5.20	4.20	5.19	c (Å)	5.19	5.19
D_av_ (nm)	19	20	26	D_av_ (nm)	10	160
c-TiN:				c-GaN:		
(mol.%)	(50)	(50)	(50)	(mol.%)	(30)	-
a (Å)	4.25	4.25	4.25	a (Å)	4.51	-
D_av_ (nm)	10	12	13	D_av_ (nm)	6	-
**Composite_950 °C**				**GaN_950 °C**		
h-GaN:				h-GaN:		
(mol.%)	(50)	(50)	(50)	(mol.%)	(80)	(100)
a (Å)	3.19	3.18	3.19	a (Å)	3.19	3.19
c (Å)	5.19	5.18	5.19	c (Å)	5.17	5.19
D_av_ (nm)	35	33	37	D_av_ (nm)	17	31
c-TiN:				c-GaN:		
(mol.%)	(50)	(50)	(50)	(mol.%)	(20)	-
a (Å)	4.24	4.24	4.25	a (Å)	4.50	-
D_av_ (nm)	12	13	14	D_av_ (nm)	7	-

**Table 3:** Vicker’s hardness H_v_ (100 and 300 g force loads) and helium density d_He_ data for composite nitride nanoceramics in the system GaN–TiN. Percentages shown in helium density data were calculated with respect to the theoretical density of 5.73 g/cm^3^ for composite h-GaN:c-TiN = 1:1 (molar basis). Note that relevant cases of individual GaN and TiN nanoceramics are included in [31] and [32], respectively.

Composite Nanoceramics From	Sintering Temperature		
	650 °C	1000 °C	1200 °C
**Composite_800**			
d_He_ (SD) (g/cm^3^)	4.43 (0.04)	4.47 (0.05)	4.48 (0.04)
% theor.	77	78	78
H_V_ (SD) (GPa):			
under 100 gf	11.3 (1.1)	11.1 (1.5)	14.1 (1.1)
**under 300 gf**	**11.1 (0.8)**	**10.0 (1.3)**	**13.2 (1.4)**
**Composite_950**			
d_He_ (SD) (g/cm^3^)	4.66 (0.03)	4.25 (0.05)	4.46 (0.03)
% theor.	81	74	78
H_V_ (SD) (GPa):			
under 100 gf	7.0 (0.7)	13.8 (1.3)	9.6 (1.2)
**under 300 gf**	**7.5 (0.5)**	**13.2 (1.1)**	**8.6 (0.9)**

## Data Availability

The data presented in this study are available on request from the corresponding author.

## References

[1] Qin R., Wang P.Y., Lin C., Cao F., Zhang J.Y., Chen L., Mu S.C. (2021). Transition metal nitrides: Activity origin, synthesis and electrocatalytic applications. Acta Phys. Chim. Sin..

[2] Ng T.K., Holguin-Lerma J.A., Kang C.H., Ashry I., Zhang H.F., Bucci G., Ooi B.S. (2021). Group-III-nitride and halide-perovskite semiconductor gain media for amplified spontaneous emission and lasing applications. J. Phys. D-Appl. Phys..

[3] Ashraf I., Rizwan S., Iqbal M. (2020). A comprehensive review on the synthesis and energy applications of nano-structured metal nitrides. Front. Mater..

[4] Dongil A.B. (2019). Recent progress on transition metal nitrides nanoparticles as heterogeneous catalysts. Nanomaterials.

[5] Nakamura S. (2015). Background story of the invention of efficient InGaN blue-light-emitting diodes (Nobel lecture). Angew. Chem. Int. Edit..

[6] Elagin A.A., Beketov A.R., Baranov M.V., Shishkin R.A. (2013). Aluminum nitride. Preparation methods. Refract. Ind. Ceram..

[7] Alhussain H., Mise T., Matsuo Y., Kiyono H., Nishikiori K., Akashi T. (2019). Influence of ammonia gas exposure on microstructure of nanocrystalline titanium nitride powder synthesized from titanium dioxide. J. Ceram. Soc. Jpn..

[8] Jiang X.F., Weng Q.H., Wang X.B., Li X., Zhang J., Golberg D., Bando Y. (2015). Recent progress on fabrications and applications of boron nitride nanomaterials: A review. J. Mater. Sci. Technol..

[9] Hirao K., Hyuga H., Hotta M., Kondo N. (2013). Review and overview of silicon nitride and SiAlON, including their applications. Handbook of Advanced Ceramics: Materials, Applications, Processing, and Properties.

[10] Levason B., Hector A.L. (2013). Chemistry and applications of metal nitrides. Coordin. Chem. Rev..

[11] Mohney S.E., Lin X. (1996). Estimated phase equilibria in the transition metal-Ga-N systems: Consequences for electrical contacts to GaN. J. Electron. Mater..

[12] Lu C.J., Davydov A.V., Josell D., Bendersky L.A. (2003). Interfacial reactions of Ti/n-GaN contacts at elevated temperature. J. Appl. Phys..

[13] Jeitschko W., Nowotny I.L., Benesovsky F. (1964). Die h-phasen: Ti_2_CdC, Ti_2_GaC, Ti_2_GaN, Ti_2_InN, Zr_2_InN und Nb_2_GaC. Monatsh. Chem..

[14] Manoun B., Kulkarni S., Pathak N., Saxena S.K., Amini S., Barsoum M.W. (2010). Bulk moduli of Cr2GaC and Ti2GaN up to 50 GPa. J. Alloy. Compd..

[15] https://www.materialsproject.org/materials/mp-1025550/.

[16] Drygaś M., Jeleń P., Radecka M., Janik J.F. (2016). Ammonolysis of polycrystalline and amorphized gallium arsenide GaAs to polytype-specific nanopowders of gallium nitride GaN. RSC Adv..

[17] Jegier J.A., McKernan S., Purdy A.P., Gladfelter W.L. (2000). Ammonothermal conversion of cyclotrigallazane to GaN:  Synthesis of nanocrystalline and cubic GaN from [H_2_GaNH_2_]_3_. Chem. Mater..

[18] Rawat V., Zakharov D.N., Stach E.A., Sands T.D. (2009). Pseudomorphic stabilization of rocksalt GaN in TiN/GaN multilayers and superlattices. Phys. Rev. B.

[19] Janik J.F., Wells R.L. (1996). Gallium imide, {Ga(NH)_3/2_}_n_, a new polymeric precursor for gallium nitride powders. Chem. Mater..

[20] Janik J.F., Wells R.L., Coffer J.L., St. John J.V., Pennington W.T., Schimek G.L. (1998). Nanocrystalline aluminum nitride and aluminum/gallium nitride nanocomposites *via* transamination of [M(NMe_2_)_3_]_2_, M = Al, Al/Ga(1/1). Chem. Mater..

[21] Coffer J.L., Waldek Zerda T., Appel R., Wells R.L., Janik J.F. (1999). Micro-Raman investigation of nanocrystalline GaN, AlN, and an AlGaN composite prepared from pyrolysis of metal amide-imide precursors. Chem. Mater..

[22] Drygas M., Olejniczak Z., Grzanka E., Bucko M.M., Paine R.T., Janik J.F. (2008). Probing the structural/electronic diversity and thermal stability of various nanocrystalline powders of gallium nitride GaN. Chem. Mater..

[23] Drygas M., Jelen P., Bucko M.M., Olejniczak Z., Janik J.F. (2015). Ammonolytical conversion of microcrystalline gallium antimonide GaSb to nanocrystalline gallium nitride GaN: Thermodynamics vs. topochemistry. RSC Adv..

[24] Drygas M., Sitarz M., Janik J.F. (2015). Ammonolysis of gallium phosphide GaP to the nanocrystalline wide bandgap semiconductor gallium nitride GaN. RSC Adv..

[25] Drygas M., Czosnek C., Paine R.T., Janik J.F. (2006). Two-stage aerosol synthesis of titanium nitride TiN and titanium oxynitride TiO_x_N_y_ nanopowders of spherical particle morphology. Chem. Mater..

[26] Stelmakh S., Grzanka E., Gierlotka S., Janik J.F., Drygas M., Lathe C., Palosz B. (2011). Compression and thermal expansion of nanocrystalline TiN. Z. Kristallogr. Proc..

[27] Borysiuk J., Caban P., Strupinski W., Gierlotka S., Stelmakh S., Janik J.F. (2007). TEM investigations of GaN layers grown on silicon and sintered GaN nano-ceramic substrates. Cryst. Res. Technol..

[28] Drygas M., Janik J.F., Gosk J., Gierlotka S., Palosz B., Twardowski A. (2016). Structural and magnetic properties of ceramics prepared by high-pressure high-temperature sintering of manganese-doped gallium nitride nanopowders. J. Eur. Ceram. Soc..

[29] Maya L. (1986). Synthetic approaches to aluminum nitride via pyrolysis of a precursor. Adv. Ceram. Mater..

[30] Brown G.M., Maya L. (1988). Ammonolysis products of the dialkylamides of titanium, zirconium, and niobium as precursors to metal nitrides. J. Am. Ceram. Soc..

[31] Drygas M., Kapusta K., Janik J.F., Bucko M.M., Gierlotka S., Stelmakh S., Palosz B., Olejniczak C. (2020). Novel nanoceramics from in situ made nanocrystalline powders of pure nitrides and their composites in the system aluminum nitride AlN/gallium nitride GaN/aluminum gallium nitride Al_0.5_Ga_0.5_N. J. Eur. Ceram. Soc..

[32] Drygaś M., Lejda K., Janik J.F., Musielak B., Gierlotka S., Stelmakh S., Pałosz B. (2021). Composite nitride nanoceramics in the system titanium nitride (TiN)-aluminum nitride (AlN) through high pressure and high temperature sintering of synthesis-mixed nanocrystalline powders. Materials.

[33] Nöth H., Konrad P. (1975). Preparation, structure and some reactions of trisdimethylaminogallane. Z. Naturforsch..

[34] Bradley D.C., Gitlitz M.H. (1969). Metallo-organic compounds containing metal-nitrogen bonds. Part VI. Infrared and nuclear magnetic resonance of dialkylamido-derivatives of titanium. J. Chem. Soc. A.

[35] Drygaś M., Bućko M.M., Musiał M., Janik J.F. (2016). Convenient synthesis of nanocrystalline powders of phase-pure manganese nitride *η*-Mn_3_N_2_. J. Mater. Sci..

[36] Patsalas P., Kalfagiannis N., Kassavetis S. (2015). Optical properties and plasmonic performance of titanium nitride. Materials.

[37] Wang L., Jiang W., Chen L., Yang M., Zhu H. (2006). Consolidation of nano-sized TiN powders by spark plasma sintering. J. Am. Ceram. Soc..

[38] Lengauer W. (1992). Properties of bulk *δ*-TiN_1-x_ prepared by nitrogen diffusion into titanium metal. J. Alloys Compd..

[39] Stoehr M., Shin C.S., Petrov I., Greene J.E. (2011). Raman scattering from TiNx (0.67 ≤ x ≤ 1.00) single crystals grown on MgO (001). J. Appl. Phys..

[40] Ponon N.K., Appleby D.J.R., Arac E., King P.J., Ganti S., Kwa K.S.K., O′Neill A. (2015). Effect of deposition conditions and post deposition anneal on reactively sputtered titanium nitride thin films. Thin Solid Film..

[41] Guo Q.X., Xie Y., Wang X.J., Lv S.C., Hou T., Bai C.N. (2005). Synthesis of uniform titanium nitride nanocrystalline powders via a reduction–hydrogenation–dehydrogenation–nitridation route. J. Am. Ceram. Soc..

[42] Bernard M., Deneuville A., Thomas O., Gergaud P., Sandstrom P., Birch J. (2000). Raman spectra of TiN/AlN superlattices. Thin Solid Film..

[43] Spengler W., Kaiser R., Christensen A.N., Muller-Vogt G. (1978). Raman scattering, superconductivity, and phonon density of states of stoichiometric and nonstoichiometric TiN. Phys. Rev..

[44] Huang Y., Chen X.D., Fung S., Beling C.D., Ling C.C. (2004). Spatial characterization of a 2 in GaN wafer by Raman spectroscopy and capacitance-voltage measurements. J. Phys. D-Appl. Phys..

[45] Pharr G.M., Herbert E.G., Gao Y.F. (2010). The indentation size effect: A critical examination of experimental observations and mechanistic interpretations. Annu. Rev. Mater. Res..

[46] Ionenaga I. (2001). Mechanical stability of power device materials high temperature hardness of SiC, AlN and GaN. Chem. Sus. Dev..

[47] Kuo C.C., Lin Y.T., Chan A., Chang J.T. (2019). High temperature wear behavior of titanium nitride coating deposited using high power impulse magnetron sputtering. Coatings.

